# Protocol for assessing enzymatic and non-enzymatic hydrogen sulfide production and levels

**DOI:** 10.1016/j.xpro.2026.104694

**Published:** 2026-07-09

**Authors:** María Ángeles Cáliz-Molina, Inmaculada Pino-Pérez, András K. Ponti, Christopher Hine, Alejandro Martín-Montalvo

**Affiliations:** 1 Andalusian Molecular Biology and Regenerative Medicine Centre-CABIMER, Universidad de Sevilla-CSIC-Universidad Pablo de Olavide, 41092 Seville, Spain; 2 Cleveland Clinic Research, Cleveland, OH 44195, USA; 3 Department of Molecular Medicine, Cleveland Clinic Lerner College of Medicine of Case Western Reserve University, Cleveland, OH 44195, USA; 4 CIBER de Diabetes y Enfermedades Metabólicas Asociadas (CIBERDEM), Instituto de Salud Carlos III, Madrid, Spain

**Keywords:** Single-molecule Assays, Cell Biology, Metabolism, Molecular Biology, Signal Transduction, Chemistry

## Abstract

Hydrogen sulfide (H_2_S) is a key molecule in metabolism with important physiological effects. Despite its merits, detection and/or accurate determination remain challenging. Here, we present a protocol to determine H_2_S levels and cumulative production using a microsulfide ion electrode and lead acetate paper. We describe steps for measurement of H_2_S levels, as well as detection of enzymatic and non-enzymatic cumulative H_2_S production across various biological contexts. This protocol is thus compatible with H_2_S donors, tissue samples, and live cultured cells.

For complete details on the use and execution of this protocol, please refer to Caliz-Molina et al.^1^

## Highlights

• Guidance on measuring H_2_S levels using a microsulfide ion electrode
• Steps to determine cumulative enzymatic H_2_S production by the lead acetate paper test
• Steps to measure cumulative non-enzymatic H_2_S production by the lead acetate paper test
• Instructions for testing H_2_S release using assays that include biological samples

## Abstract (editor highlights)

Publisher’s note: Undertaking any experimental protocol requires adherence to local institutional guidelines for laboratory safety and ethics.

## Graphical abstract

**Figure:**
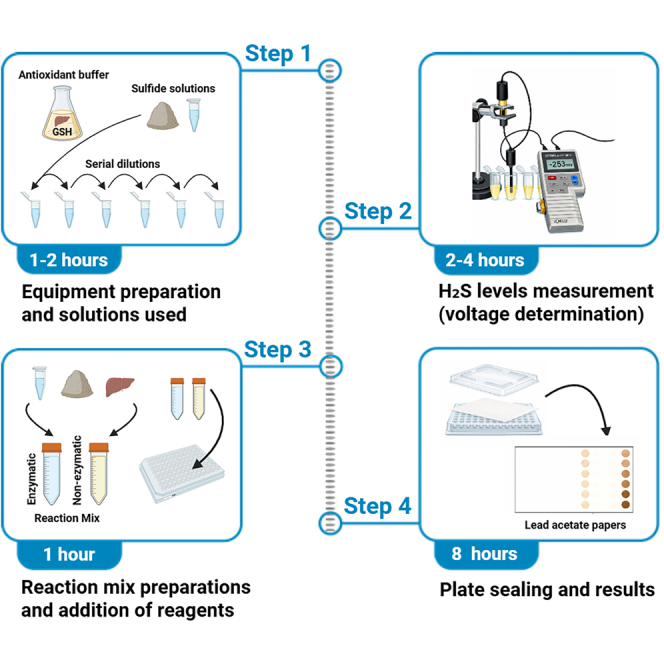

## Before you begin

The following protocol comprises two distinct workflows within a single methodological framework, each designed to address a specific analytical objective related to H_2_S levels and production capacity. Workflow 1 focuses on the quantification of H_2_S levels using a microsulfide ion electrode (Arrow H_2_S™), that measures sulfide in solution under highly alkaline conditions (pH > 13.3), where sulfide is predominantly present as S^2-^ in equilibrium, and minimizes volatilization and oxidation. Workflow 2 is designed to determine the semi-quantitative cumulative production of H_2_S over a defined incubation time, including both enzymatic and non-enzymatic contributions, using a lead acetate paper test.

Please note these techniques differ: one measures H_2_S concentration (by determining sulfide ion S^2-^) with a microsulfide ion electrode, whereas the other one determines cumulative H_2_S production over a defined incubation period semi-quantitatively using a lead acetate test, in which colourless lead acetate impregnated onto filter paper reacts with H_2_S gas to form lead sulfide, resulting in a darkened coloration. The intensity of the dark colour provides an indirect measure of the cumulative H_2_S production over time.

For clarity, the protocol is organized into two sections corresponding to these workflows: Workflow 1 (Steps 1–6) describes the measurement of H_2_S levels using the microsulfide ion electrode, while Workflow 2 (Steps 7–13) details the determination of cumulative H_2_S production by the lead acetate paper test. The two workflows belong to the same protocol, but can be performed either independently or in combination, depending on the experimental objective. You may therefore follow the entire protocol to determine both H_2_S levels and cumulative production or select only the workflow relevant to your experiment.

More specifically, the examples below describe the specific steps for measuring H_2_S levels and the accumulative non-enzymatic production of H_2_S from natural sulfur-enriched compounds found in garlic cloves, such as diallyl disulfide (DAD) and diallyl trisulfide (DAT).^1^ In several assays, to emulate the effect of abundant cellular thiols that are proposed to reduce these sulfur-enriched compounds and promote H_2_S release, we added reduced glutathione (GSH). We also included liver lysates as a biological system. Furthermore, we measured the enzymatic H_2_S production in liver tissue from mice fed with these compounds. In our experience, we have applied this protocol to other tissues, such as human plasma.^1^ This method to determine H_2_S production can also be applied to living systems, such as cultured cells (for example, AML12 cells^1^ and yeast^2^).

### Innovation

As H_2_S is a gaseous molecule, its capture, and/or accurate detection present significant challenges, and developing optimal methods for these purposes has proven extremely difficult within the scientific community. However, this protocol introduces a flexible and question-driven framework for H_2_S assessment and detection by integrating two complementary and methodologically distinct approaches within a single protocol. Rather than proposing a one-size-fits-all solution, the innovation lies in guiding researchers toward the most appropriate method based on their specific experimental objectives and expected outcomes.

In workflow 1, we provide a detailed and practical protocol for H_2_S quantification using an microsulfide ion electrode (ArrowH_2_S™), including critical troubleshooting steps and, innovatively, the incorporation of visual guidance through pictures and videos to facilitate reproducibility and reduce common experimental errors. We developed this protocol to be able to measure biological samples, such as tissue lysates in the presence of other biological substrates, such as GSH. Using this approach, we determine levels of H_2_S present in solution that can be reliably measured while recreating biologically relevant environments.

In workflow 2, we extend beyond traditional enzymatic assays by developing a comprehensive protocol that determines semi-quantitatively cumulative H_2_S production from both enzymatic and non-enzymatic sources, using the lead acetate test. We do so by introducing alternative substrates, including guidance to determine H_2_S generation in the presence of biological samples, such as tissue lysates and cell cultures, thereby broadening the applicability of enzymatic and non-enzymatic H_2_S production assays to highly physiologically relevant contexts. It should be noted that the lead acetate test provides a relative assessment of H_2_S production capacity compared with a control condition. Signal intensity can be evaluated by computer-assisted densitometric analysis of digital images of the resulting lead sulfide spots on the lead acetate paper.

### Institutional permissions

CABIMER Animal Committee approved mice experiments and we performed these experiments in accordance with the Spanish law on animal use RD 53/2013 and the EU Directive (2010/63/EU) for animal research.

### Preparation for measurement of H_2_S levels using an ion-selective microelectrode (workflow 1)

**Timing: 1–2 h**

**Figure 1:**
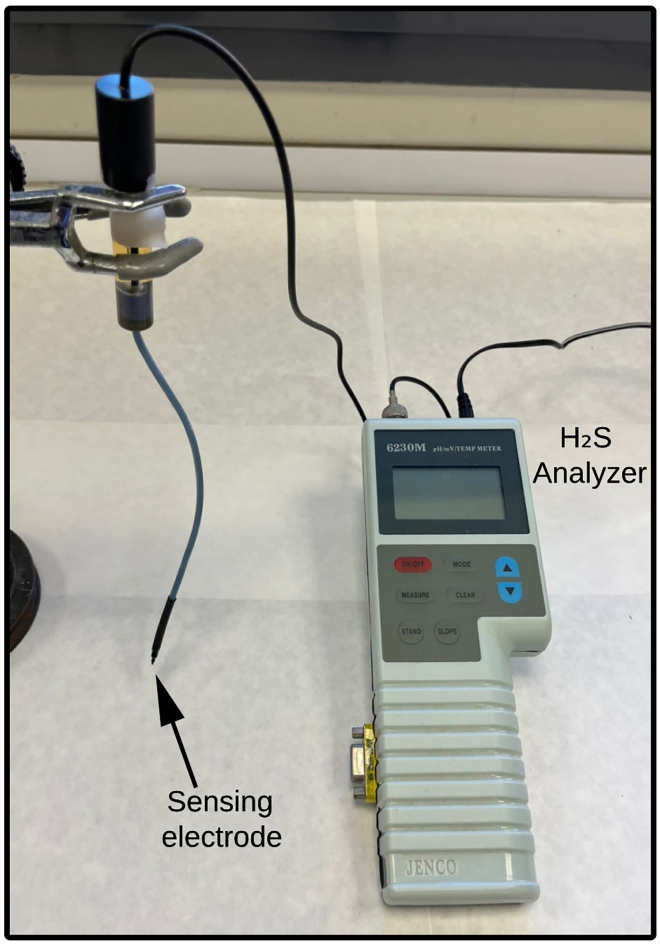
ArrowH_2_S™ H_2_S MEASUREMENT SYSTEM The H_2_S measurement system consists of the ArrowH_2_S™ micro sensing electrode and the Model 6230N H_2_S analyzer, used together for accurate detection and quantification of H_2_S.

The ArrowH_2_S™ H_2_S MEASUREMENT SYSTEM (Model No. ISM-146H2S-XS) consists of two parts; the ArrowH_2_S™ micro sensing electrode; and the Model 6230N H_2_S analyser (Figure 1).

**CRITICAL:** Prepare all reagents in a chemical fume hood while maintaining a consistent temperature (*∼*25 °C) to ensure safe handling and minimize the loss of volatile compounds such as H_2_S. Keep solutions in tightly capped tubes whenever possible and open them only during assays preparation and measurements to reduce volatilization and air exposure.

**CRITICAL:** Avoid any contact between the electrode sensing tip and the skin.

***Note:*** Direct any technical questions about operating the electrode to Lazar Research Laboratories at techsupport@lazarlab.com.

**Figure 2:**
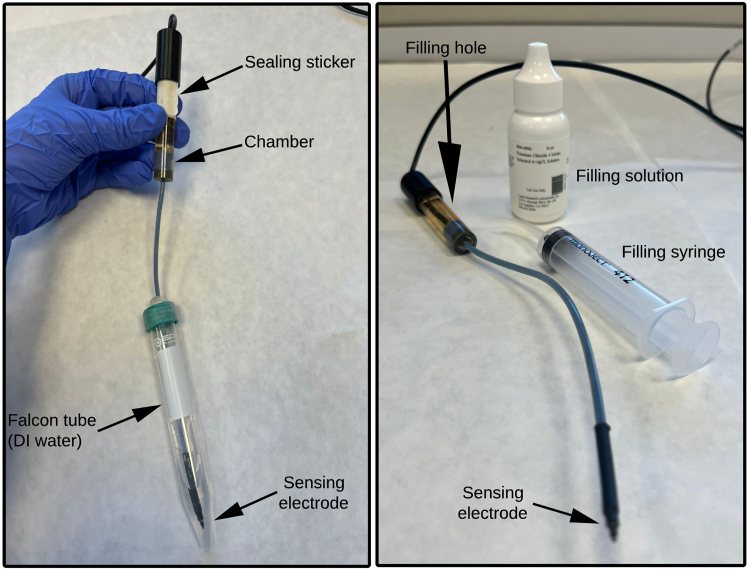
Detailed ArrowH_2_S™ micro sensing electrode The figure shows the filling solution and the syringe used to load it, the filling hole, the sensing electrode used to measure H_2_S and illustrates how the electrode is properly sealed.
  c. Invert the chamber to fill the entire electrode.
  d. Seal the hole with the provided sticker (or tape) as soon as you finish filling the electrode with the filling solution and return it to the upright position.

1. Electrode set up.
  a. Fill the ArrowH_2_S™ microsulfide ion electrode with KCl (4 M) saturated with AgCl-filling solution (this solution is supplied with the instrument).
  b. Insert the filling solution with a syringe (supplied with the instrument) into the filling hole of the chamber of the electrode (Figure 2).

**CRITICAL:** The electrode must always remain hydrated in a suitable liquid. To prevent drying, keep the electrode in a Falcon tube with distilled water and replace the water monthly.

**CRITICAL:** Proper filling of the electrode is essential. Before each use, fill the electrode completely through the opening at the top of the electrode using a filling syringe. Avoid any air bubble. Troubleshooting 1.

***Note:*** For the entire protocol, see the “materials and equipment” section for the recipes of the buffers used.

2. Prepare daily a fresh 4 × concentrated antioxidant buffer.

***Note:*** Use the antioxidant buffer to prepare Na_2_S solutions. Due to its composition, it prevents air oxidation of sulfide ions while adjusting the pH of standards and samples. Prepare this buffer fresh daily.

3. Prepare a Na_2_S standard solution.
  a. To make a 10 mM Na_2_S standard solution, dissolve 0.0024 g of sodium sulfide crystals (Na_2_S·9H_2_O) in 250 μL of 4 × concentrated antioxidant buffer.
  b. Add deionized water to bring the total volume to 1 mL.
  c. Mix the solution thoroughly.

***Note:*** Prepare this standard fresh and use it within 6 h.

**CRITICAL:** Handle chemicals with gloves and perform all pipetting in a fume hood to prevent exposure to H_2_S gas.

4. Prepare 1 × antioxidant buffer (Sodium salicylate 390,25 mM, Ascorbic acid 92,25 mM, and NaOH 531,25 mM, pH ∼13.36).
  a. Dilute the 4 × concentrated antioxidant buffer with deionized water (1 part of 4 × concentrated antioxidant buffer to 3 parts of water).
  b. Stir the solution thoroughly.

***Note:*** Prepare this buffer fresh daily.

***Note:*** Use the 1 × antioxidant buffer to make the serial dilutions described in the following points 5–7.

**Figure 3:**
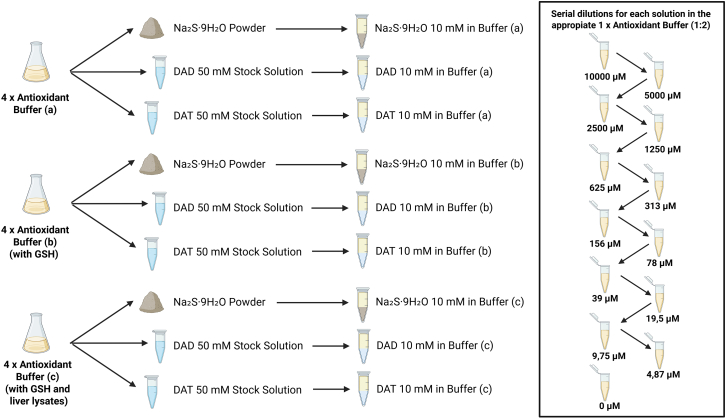
Schematic description of the solutions used Prepare each 4 × antioxidant buffer with or without GSH and liver lysates. The 4 x antioxidant buffer (a) consisted of sodium salicylate, ascorbic acid and NaOH, the 4 x antioxidant buffer (b) was the antioxidant buffer a supplemented with GSH, and the 4 x antioxidant buffer (c) was the antioxidant buffer a supplemented with GSH and liver lysates. Next, in each 4 × antioxidant buffer (a, b, and c) add Na_2_S, DAD or DAT, and add water to reach a 10 mM final concentration to generate a 1 × antioxidant buffer. Finally, make serial dilutions from each of these solutions using each specific 1 × antioxidant buffer.

***Note:*** Prepare these standards fresh and use them within 6 h.

5. Prepare additional Na_2_S standard solutions at lower concentrations. Prepare a series of Na_2_S standard solutions by stepwise diluting the 10 mM Na_2_S standard solution with 1 × antioxidant buffer to obtain concentrations ranging from 10 mM to 0 μM (Figure 3).

6. Prepare the experimental samples.
  a. Prepare these solutions in 250 μL of 4 × concentrated antioxidant buffer.
  b. Add deionized water to bring the total volume of 1 mL.
  c. Mix the solution thoroughly.
    ***Note:*** Prepare these samples fresh and use them within 6 h.
    ***Note:*** For example, to measure H_2_S levels produced from natural sulfur-enriched compounds found in garlic cloves, specifically DAD and DAT, prepare the following 4 × antioxidant buffer formulations:
  d. 4 × antioxidant buffer (a).
  e. 4 × antioxidant buffer with GSH (b): Base formulation of the 4 × antioxidant buffer with a concentration of 50 mM GSH.
  f. 4 × antioxidant buffer with GSH and liver lysates (c): Base formulation of the 4 × antioxidant buffer with a concentration of 50 mM GSH and liver homogenate (100 mg/mL).
7. Prepare additional experimental sample solutions at lower concentration. Prepare a series of diluted sample solutions by stepwise diluting the experimental samples with 1 × Antioxidant buffer to obtain concentrations ranging from 10 mM to 0 μM.

***Note:*** Prepare these samples fresh and use them within 6 h.

**Figure 4:**
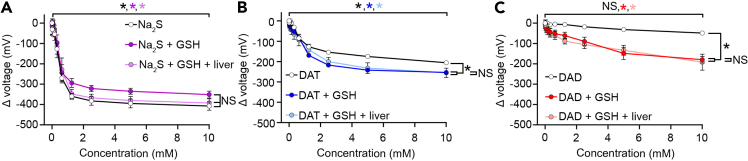
Representative Na_2_S calibration curve and illustrative examples of compounds capable of increasing H_2_S levels (A) H_2_S levels generated from Na_2_S alone, in the presence of GSH (12.5 mM), or with GSH (12.5 mM) with liver lysates (25 mg). n = 3 biological replicates. Two-way ANOVA with Tukey’s post hoc test. (B) H_2_S levels generated from DAT alone, in the presence of GSH (12.5 mM), or with GSH (12.5 mM) with liver lysates (25 mg). n = 3 biological replicates. Two-way ANOVA with Tukey’s post hoc test. (C) H_2_S levels generated from DAD alone, in the presence of GSH (12.5 mM), or with GSH (12.5 mM) with liver lysates (25 mg). n = 3 biological replicates. Two-way ANOVA with Tukey’s post hoc test. Data shown are the means ± SEM. ^∗^p < 0.05. Figure reprinted and adapted with permission from Caliz-Molina et al., 2025.^1^

***Note:*** For example, to generate 1 × antioxidant buffers (a, b, and c), dilute the 4 X antioxidant buffer solutions described above (a, b and c) with deionized water (1 part of 4 × concentrated antioxidant buffers to 3 parts of water) (Figures 4A-4C). To generate 1 × antioxidant buffers (a, b, and c) containing DAD or DAT prepare a 50 mM stock solution of DAD or DAT. Make a 10 mM working solution by mixing 200 μL of the 50 mM stock solution with 250 μL of 4 X antioxidant buffers (a, b, and c), respectively. Then, add deionized water to each vial to bring the total volume to 1 mL. Mix the solutions thoroughly. Then, make serial dilutions (1:2) ranging from 10 mM to 0 mM in 1 X antioxidant buffers (a, b, and c), respectively.

### Lead acetate paper preparation (workflow 2)

**Timing: 24 h**

8. Cut blotting paper, such as Whatman™ GB003, to approximately the size of the plate used.
9. Prepare a 20 mM lead acetate solution, dissolving 0.759 g of lead acetate trihydrate in 100 mL of distilled water.

***Note:*** We prepared the solution and use it immediately.

**CRITICAL:** Perform all steps, including weighing and handling of the chemical, while wearing an appropriate respiratory protection mask and gloves. This step and all subsequent steps must be carried out inside a fume hood.

10. Immerse the cut papers in the 20 mM lead acetate solution in an opaque glass container for approximately 20 min with gentle agitation in a shaker (e.g., 50 RPM in a standard orbital shaker). At this time, turn on the vacuum oven at 110°C.

**CRITICAL:** As lead acetate is photosensitive, the container must be opaque. Alternatively, protect the papers from light by wrapping the container containing the papers and the lead acetate solution with aluminium foil.

11. Remove the papers from the solution and dry them in the vacuum oven at 110 °C with the vacuum turned on for 20 min to ∼70–75 kPa.

***Note:*** Papers should be completely dry prior to proceeding. If a vacuum oven in not available, then alternative methods such as a hair dryer can be used, altough paper preparation is more time consuming. When using a hair dryer, ensure that the paper is dried uniformly and completely from multiple angles to prevent uneven formation of lead sulfide spots.

12. Store the dry papers at 20–22°C in a dark, dry place until use.

**Figure 5:**
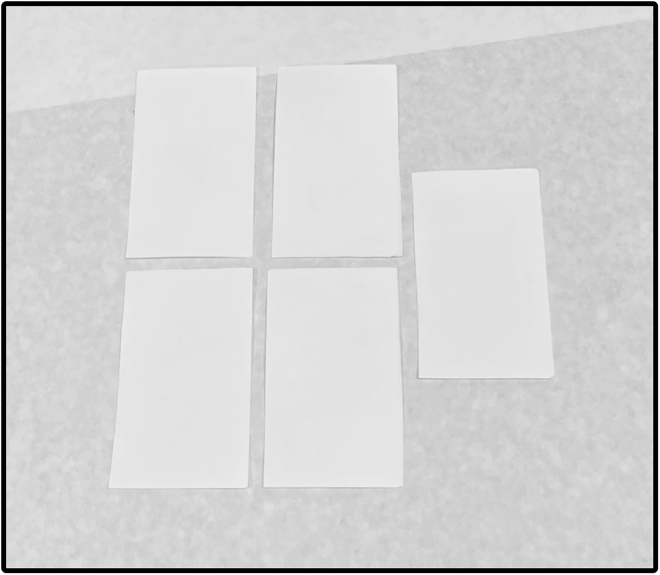
Ready-to-use lead acetate papers Prepare papers by cutting them to fit a 96-well plate format, soaking them in a lead acetate solution, allow them to dry in a vacuum oven, and ensure they remain wrinkle-free prior to use.

***Note:*** If the papers are not flat, place the papers in the fume hood (kept dark) and place a flat weight (e.g. 2 kg) on top of them for 24 h to facilitate proper flattening of the papers (Figure 5).

***Note:*** Lead acetate-impregnated papers should be used within one month.

**CRITICAL:** It is important to avoid lead acetate contact with the skin and handling lead acetate-impregnated papers with bare hands. Gloves must always be used.

### Sealing system preparation (workflow 2)

**Timing: 30 min**

The sealing system consists of a lid for a 96-well plate in which we make a hole in each well to help prevent condensation of the reaction mix. Condensation can cause the paper to become damp, which leads to it warping and/or inaccurate capture and visualization of H_2_S.

13. To prepare the lid:
  a. Light a Bunsen burner.
  b. Heat the tip of a metal needle in the burner.
  c. Insert the tip into the center of each well to make a small hole.

**CRITICAL:** When making the hole do it with caution to avoid cracking the plate.

***Note:***Methods video S1 shows a prepared lid.

Methods video S1. Proper sealing of the paper to a 96-well plate, related to “Before you begin” section

### Enzymatic solution preparation (workflow 2)

**Timing: 30 min**

**CRITICAL:** Prepare all reagents in a chemical fume hood while maintaining a consistent temperature (*∼*25°C) to ensure safe handling and minimize the loss of volatile compounds such as H_2_S. Keep solutions in tightly capped tubes whenever possible and open them only during assays preparation and measurements to reduce volatilization and air exposure.

***Note:*** The procedures described below are optimized for a 96-well plate format.

14. Prepare stock solutions of desired substrates, cofactors and inhibitors.

**Figure 6:**
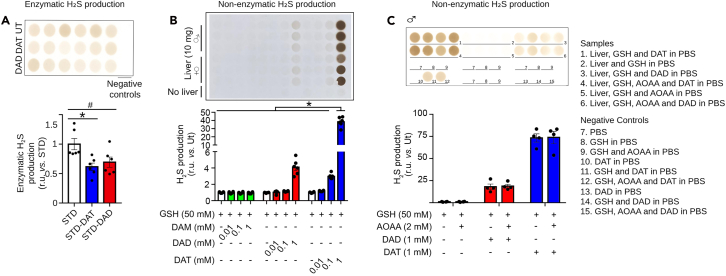
Illustrative enzymatic and non-enzymatic production of H_2_S (A) Analysis of H_2_S generation liver lysates of mice fed with standard diet untreated or supplemented with DAT or DAD with a standard healthy diet (35 μg protein/well). 2.5 h of incubation. n = 6 biological replicates. One-way ANOVA Tukey. Negative controls are PLP 1 mM and L-cysteine 10 mM without liver proteins. (B) Non-enzymatic H_2_S generation from DAM, DAD and DAT with GSH (50 mM) and liver lysates (10 mg). *n*= 6 biological replicates. 16 h of incubation. Two-way ANOVA Tukey. Negative controls are liver lysates in 50 mM GSH without H_2_S donors. (C) Non-enzymatic H_2_S generation DAD and DAT in the presence of AOAA. Controls are depicted in the figure. 7: PBS, 8: GSH in PBS: 9 GSH and AOAA in PBS. 10: DAT in PBS: 11 GSH and DAT in PBS. 12: GSH, AOAA and DAT in PBS. 13: DAD in PBS. 14: GSH and DAD in PBS. 15: GSH, AOAA and DAD in PBS. GSH: 50 mM GSH. AOAA: 2 mM AOAA. DAD: 1 mM DAD. DAT: 1 mM DAT. n = 4 biological replicates. 24 hour of incubation. Two-way ANOVA Tukey. Negative controls are liver lysates in their respective reaction mix without H_2_S donors. Data shown are the means ± SEM. ^∗^p < 0.05. Figure reprinted and adapted with permission from Caliz-Molina et al., 2025.^1^

***Note:*** Prepare enzymatic reaction mix fresh daily. Prepare L-cysteine, DL-homocysteine and mercaptopyruvate 100 mM in phosphate buffered saline (PBS) fresh daily for each experiment. Prepare a pyridoxal 5′-phosphate (PLP) stock solution by adding the PLP powder to PBS, warm to 37°C, and vortex until fully dissolved. Then, adjust to the desired final volume, and store the stock solution in aliquots at −20°C (use PLP solution within a year).

***Note:*** For example, in the experiment outlined in this protocol, the initial stocks solutions used for enzymatic production are L-cysteine 100 mM as substrate and PLP 10 mM as cofactor (Figure 6).

15. Prepare the enzymatic reaction mix by adding the desired substrates, cofactors and/or inhibitors in PBS to reach the final concentrations when samples are added.

***Note:*** The final reaction volume should not exceed 200 μL per well in a 96-well plate. 100 to 150 μL reaction volumes tend to provide best results and limit condensation and/or the liquid phase to contact with the above lead acetate filter paper.

**CRITICAL:** Keep the enzymatic reaction on ice and use it immediately.

### Non-enzymatic solution preparation (workflow 2)

**Timing: 30 min**

**CRITICAL:** Prepare all reagents in a chemical fume hood while maintaining a consistent temperature (*∼*25°C) to ensure safe handling and minimize the loss of volatile compounds such as H_2_S. Keep solutions in tightly capped tubes whenever possible and open them only during assays preparation and measurements to reduce volatilization and air exposure.

***Note:*** This is an optimized procedure for a 96-well plate format.

16. Prepare stock solutions compounds evaluated for non-enzymatic H_2_S production.
  ***Note:*** For example, in the experiment described in this protocol, we investigated whether naturally occurring sulfur-enriched compounds such as diallyl monosulfide (DAM), DAD and DAT generate H_2_S.
  a. We prepared fresh DAM (100 mM), DAD (100 mM) and DAT (100 mM) as stock solutions.
  b. Additionally, we used a fresh daily prepared 163 mM GSH solution as stock solution in the reaction mix to enhance non-enzymatic H_2_S production.
17. Prepare the non-enzymatic reaction mix by adding the substrates and/or inhibitors in PBS.
  ***Note:*** For example, in the experiments described in Figure 6B, the non-enzymatic reaction mix contains 100 mM GSH in PBS (100 μL/well). In experiments shown in Figure 6C, we prepared 3 reaction mixes:
  a. PBS (100 μL/well).
  b. 100 mM GSH in PBS (100 μL/well). The final reaction volume is 200 μL per well in a 96-well plate.
  c. 100 mM GSH in PBS with aminooxyacetic acid (AOAA) 4 mM (100 μL/well). The final reaction volume is 200 μL per well in a 96-well plate.
    **CRITICAL:** Keep the non-enzymatic reaction mix on ice and use immediately.
    ***Note:*** Additional substrates can be incorporated into the reaction mixture to evaluate different mechanisms of non-enzymatic H_2_S production, including proteinase K–pretreated samples, Fe^3+^, PLP, and D-cysteine.^3^

## Key resources table

**Table:** 

REAGENT or RESOURCE	SOURCE	IDENTIFIER
**Biological samples**		

Mouse liver tissue of C57BL/6J Wt mice (females and males, 4–40 weeks of age)	Charles River	RRID:IMSR_JAX:000664

**Chemicals, peptides, and recombinant proteins**		

Diallyl disulfide	LKT Laboratories	A4544
Diallyl trisulfide	LKT Laboratories	D3202
Diallyl monosulfide	Sigma-Aldrich	A35801
Na_2_S∗9H_2_0	Sigma-Aldrich	387065000
Lead (II) acetate trihydrate	Sigma-Aldrich	316512
L-Cysteine	Sigma-Aldrich	168149
Pyridoxal 5′-phosphate hydrate	Sigma-Aldrich	P9255
L-Glutathione reduced	Sigma-Aldrich	G6013
5x passive lysis buffer	Promega	E1941
Sodium salicylate	Thermofisher	A17056
Ascorbic Acid	Sigma-Aldrich	A92902
Sodium hydroxide	Sigma-Aldrich	1.06498.0500
AOAA	TargetMol	T5880
DL-Homocysteine	Sigma-Aldrich	H4628
L-Homocysteine	Sigma-Aldrich	69453
Sodium mercaptopyruvate	Santa Cruz Biotechnology	sc-236908

**Experimental models: Organisms/strains**		

C57BL/6J Wt mice (females and males, 4–40 weeks of age)	Charles River	RRID:IMSR_JAX:000664

**Software and algorithms**		

Graph Pad Prism 7	Graphpad	RRID:SCR_002798
ImageJ	NIH	RRID:SCR_001935

**Other**		

96 well microplate	Greiner	655101
Vacuum oven	VWR	89508-424
Incubator	Thermo Scientific	51028133
D1000 Handheld Homogenizer	Sigma-Aldrich	Z742488
Protein Cleaning Solution	Thermo Scientific	ECDPCBT
GB003 Filter paper	Whatman	10426892
Blotting paper	VWR	28298-026

## Materials and equipment

**Table:** 4 × concentrated antioxidant buffer

Reagent	Final concentration	Amount
Sodium salicylate	1561 mM	25 g
Ascorbic acid	369 mM	6.5 g
NaOH	2125 mM	8.5 g
Total	–	100 ml

Storage conditions: This buffer should be made fresh daily.

**Table:** Serial dilutions of Na_2_S and experimental samples

Tube	Volume transferred from previous tube (μL)	Diluted antioxidant buffer (μL)	Dilution factor	Final concentration (μM)
1	10 mM stock solution	—	—	10000
2	500	500	1:2	5000
3	500	500	1:2	2500
4	500	500	1:2	1250
5	500	500	1:2	625
6	500	500	1:2	313
7	500	500	1:2	156
8	500	500	1:2	78
9	500	500	1:2	39
10	500	500	1:2	19,5
11	500	500	1:2	9,75
12	500	500	1:2	4,87
13	—	1000	—	0

Storage conditions: These solutions should be used within 6 h.

**Table:** Main commonly used substrates, cofactors and inhibitors of Enzymatic H_2_S Production

Compound	Role in H_2_S production	Final concentration
L-Cysteine	Cystathionine β-synthase and Cystathionine γ-lyase substrate	10 mM
DL-Homocysteine or L-Homocysteine	Cystathionine γ-lyase substrate and Cystathionine β-synthase substrate when combined with L-cysteine	10 mM
Sodium mercaptopyruvate	3-mercaptopyruvate sulfurtransferase substrate	10 mM
PLP	Cystathionine β-synthase and Cystathionine γ-lyase cofactor	1 mM
AOAA	Inhibitor of PLP-dependent enzymes	2 mM

Storage conditions: Cysteine, Homocysteine and mercaptopyruvate solutions should be made fresh daily. PLP and AOAA stock solutions can be frozen, stored at −20 °C and used within a year.

## Step-by-step method details

***Note:*** Scientists who wish to perform both workflows should follow the complete sequence of steps (Steps 1–13). Those interested in only one workflow should follow the step range corresponding to the selected workflow (steps 1 to 6 to determine H_2_S levels or 7 to 13 to determine H_2_S cumulative production).

### Measurement of H_2_S levels using an ion-selective microelectrode

**Timing: 2**–**4 h** (Depending on the number of dilutions and reagents to be tested).

Here, we detail the steps for H_2_S quantification in biological samples using an H_2_S ion-selective microelectrode, including tissue lysates and biological substrates such as GSH.

1. Connect ArrowH_2_S™ micro electrode to Model 6230N analyzer. Switch H_2_S analyzer to read millivolts (mV).
2. Rinse the electrode with distilled water and place the electrode in 10 mM Na_2_S standard solution for 30 min.
3. Place the electrode into 1 × antioxidant buffer and record the stabilized mV reading (∼5 min maximum).

***Note:*** To ensure optimal electrode performance and minimize experimental variability, all measurements should be conducted at a consistent temperature of *∼*25°C within a chemical fume hood.

**CRITICAL:** Rinse the electrode thoroughly with deionized water before transferring it to the next concentration of the standard or unknown samples.

**CRITICAL:** Check the sensitivity of the electrode every 2 h by placing the electrode in a fresh aliquot of 1 × antioxidant buffer. If the values deviate by more than 4% from the original value of 1 × antioxidant buffer**,** place the electrode in 10 mM Na_2_S standard solution for 30 min and measure the Na_2_S standards again.

4. Measure each standard and samples sequentially.

**CRITICAL:** To minimize experimental variability, all measurements should be performed under strictly standardized conditions, including reaction volume, incubation time, and temperature. Unnecessary sample agitation and delays prior to measurement should be avoided, as H_2_S degradation and volatilization may adversely affect signal intensity and reproducibility.

***Note:*** When using biological samples, the manufacturer recommends remove proteins from the sample by ultrafiltration (10 kDa cut off filter) to extend the life of the microelectrode. Troubleshooting 2.

***Note:*** Once you complete the protocol, store the electrode immersed in distilled water.

5. Subtract the mV values of the 1 × antioxidant buffer (0 μM solution) a, b and c to the readings on each corresponding standard or sample. Plot the decrease on mV values on a linear Y-axis against the corresponding Na_2_S concentration (X-axis), covering the range from 10 mM to 0 μM (Figure 4).
6. Apply the same procedure to the experimental samples.

***Note:*** The concentration of H_2_S in unknown samples can be estimated indirectly using the Na_2_S calibration curve generated under identical experimental conditions. Specifically, the millivolt (mV) response obtained from the sulfide ion-selective electrode for each unknown sample should be interpolated against the Na_2_S standard curve to determine the corresponding sulfide concentration.^4^

***Note:*** For example, we studied the capacity of naturally occurring sulfur-enriched compounds from garlic cloves, DAD and DAT to generate H_2_S levels in solution. Figure 5A displays the standard curves of Na_2_S generated in 1 × antioxidant buffer a, 1 × antioxidant buffer b, which is supplemented with 12.5 mM GSH, and 1 × antioxidant buffer c, which is supplemented with 12.5 mM GSH and liver lysates (25 mg). Figure 4B and 4C shows a dose-dependent increase in H_2_S levels promoted by DAT and DAD. DAT solutions release detectable H_2_S levels in 1 × antioxidant buffer a, b and c. DAD required the presence of GSH (1 × antioxidant buffer b) to significantly generate H_2_S levels. The presence of liver lysates (1 × antioxidant buffer c) in the assay did not alter H_2_S levels in these solutions.

### Determination of cumulative enzymatic and non-enzymatic H_2_S production by the lead acetate paper test

**Timing: 24–48 h**

Here, we detail the step-by-step procedure to determine semi-quantitatively the cumulative H_2_S production from both enzymatic and non-enzymatic sources using the lead acetate test.

7. Homogenate biological samples in lysis buffer, reaction mix or PBS and add lysates to the designated wells of a 96-well plate.

***Note:*** For example, we used a D1000 Handheld Homogenizer in the experimental example to homogenize liver tissue.

***Note:*** Liver protein content can be quantified to ensure that an equal amount of protein is loaded in all wells.

8. Add the remaining substrates of the enzymatic or non-enzymatic reactions to the plate and the H_2_S donor. Using a 96 well plate, ensure that the final volume in the assay must not exceed 200 μL.

**CRITICAL:** Include appropriate controls in the reaction to ensure proper interpretation of the results. For enzymatic H_2_S production, add the reaction mix lacking liver homogenate and add the tissue lysis buffer (*e.g.* 1x passive lysis buffer) to reach the same final volume. For non-enzymatic H_2_S production, add the non-enzymatic reaction mix lacking H_2_S donors and add the vehicle used to generate H_2_S donors to reach the same final volume. The pH of the reaction mixture may influence both catalytic activity and H_2_S release. As these reactions are typically performed in buffered saline solutions at near-physiological pH (*∼*7.2–7.4), sulfide is expected to be present predominantly as a mixture of HS^-^ and H_2_S. Because the lead acetate paper assay detects only volatile species diffusing from the gas phase, without direct contact between the liquid phase and the filter paper, signal generation primarily reflects volatile sulfide species capable of reacting with lead acetate to form lead sulfide spots. Although reaction pH may be adjusted to shift sulfide speciation and enrich specific sulfide forms, pH values outside the physiological range may alter reaction kinetics or interfere with assay performance, particularly in reactions involving protein enzymes and amino acid substrates.

***Note:*** To evaluate the applicability of this method to additional biological systems, our previous work included measurements of H_2_S production in AML12 cells and from plasma samples obtained from *in vivo* mouse experiments.^1^ Earlier studies also demonstrated that this method can be used to determine H_2_S-production capacity across a variety of biological samples including yeast, whole fly lysates and whole worm lysates, and rodent tissue lysates such as kidney.^2^

**CRITICAL:** Make sure there is no residual or spilled liquid on the rims of the wells of the plate. If needed, gently remove it with a lint-free tissue. Troubleshooting 3.

***Note:*** For example, in the experiment shown in Figure 6A, we tested enzymatic H_2_S producion in liver extracts. We added the reaction mix (125 μL) to the 96 well plate and then we added liver protein isolations in 1× passive lysis buffer (25 μL) to reach a final volume of 150 μL (final concentrations 10 mM cysteine and 1 mM PLP). As negative control, we added the enzymatic mix (125 μL) and 25 μL of passive lysis buffer (Figure 6A). Negative controls are PLP 1 mM and L-cysteine 10 mM without liver proteins.

In the experiment shown in Figure 6B, we tested non-enzymatic H_2_S producion in garlic compounds and the effect or liver lysates in the production. We lysed the livers in the non-enzymatic reaction mix (100 mM GSH in PBS). We added the non-enzymatic reaction mix (containing liver lysates or not) (100 μL/well). Finally, we added DAM, DAD, DAT or vehicle (from 0 to 1000 μM final concentration) (100 μL/well). The final concentration of GSH is 50 mM. Negative controls are liver lysates in 50 mM GSH with vehicle.

In experiments shown in Figure 6C, we added 3 reaction mixes to the plate, 1) PBS, 2) 100 mM GSH in PBS and 3) 100 mM GSH in PBS with AOAA 4 mM (100 μL/well in all cases). Then, we added liver lysates in PBS (50 μL/well). Finally, we added DAD, DAT or vehicle (50 μL/well). The final concentrations were GSH (50 mM), AOAA (2 mM), DAD (0 or 1 mM) and DAT (0 or 1 mM) (200 μl/well). Negative controls are liver lysates in 50 mM GSH with vehicle. As additional controls, we added the three reaction mixes without adding liver lysates, maintaining the same final concentrations of all reagents (200 μL/well final volume). 7: PBS, 8: GSH in PBS: 9 GSH and AOAA in PBS. 10: DAT in PBS: 11 GSH and DAT in PBS. 12: GSH, AOAA and DAT in PBS. 13: DAD in PBS. 14: GSH and DAD in PBS. 15: GSH, AOAA and DAD in PBS (Figure 6C).

9. Gently place the dry lead acetate–impregnated paper on top of the 96-well plate, ensuring full coverage.
10. Secure the lead acetate paper to prevent any movement during the procedure.
  a. Place a rigid plastic cover (the plate lid with holes prepared in “before you begin” section) over the paper.
  b. Fix it in place with tape or a heavy weight such as a centrifuge tube heat block.
  c. Ensure direct contact with the rims of the wells.

***Note:*** It is essential to secure the paper firmly to the plate. Otherwise, the evaporation may lift the paper, preventing proper formation and clear edges of the lead sulfide circles.

**CRITICAL:** Perform the assay using a tightly assembled 96-well plate system (plate, lead acetate paper and cover) to avoid H_2_S loss during incubation. Ensure that lead acetate paper is securely positioned between the plate and cover to create a semi-sealed environment with limited headspace above the reaction mixture while avoiding direct contact between the liquid sample and the lead acetate paper. Maintain consistent incubation conditions, including temperature, incubation time, and plate positioning, and avoid opening or disturbing the plate during incubation, as this may result in H_2_S loss and artifacts.

11. Transfer the sealed plate into a 37°C incubator.
12. After 30 min and at subsequent time points, observe the paper without displacing it from its position by examining underneath: this is why a clear 96 well plate works best. Dark circles will appear over the wells, indicating the formation of lead sulfide from the reaction between lead acetate and H_2_S (Figure 6).

**CRITICAL:** Allow the circles to develop until they are clearly visible, but avoid letting them become saturated, as this will compromise accurate image analysis. Troubleshooting 4.

***Note:*** Signal output depends on the H_2_S-producing compound, incubation time, and sample mass. To ensure reproducibility, maintaining consistent experimental conditions (including protein concentration, reaction volume, and incubation time) and optimizing incubation times to achieve detectable but non-saturated signals.

***Note:*** Incubation period varies depending on the assay (enzymatic of non-enzymatic), biological sample to be assayed and sample mass. Enzymatic H_2_S cumulative production is typically assessed over 1–4 h when using liver tissue, which has high H_2_S production capacity. Non-enzymatic reactions normally require longer incubation time (24–48 h).

***Note:*** We recommend using separate plates for enzymatic and non-enzymatic solutions. We also recommend performing several plates simultaneously to obtain measurements at different time points. Troubleshooting 5.

***Note:*** Keep the lead acetate paper protected from light and scan or photograph it immediately after completion of the incubation period, as prolonged exposure to light and ambient conditions may result in fading or loss of the dark precipitate.

**CRITICAL:** Do not handle the lead acetate paper with bare hands.

13. Signal output is quantified from images by measuring the integrated color intensity of the circles using ImageJ or equivalent image analysis software.
  a. Signal intensity is expressed as relative Integrated Density values after subtraction of the appropriate negative controls: the enzymatic mix with lysis buffer for enzymatic production and liver lysates in GSH with vehicle for non-enzymatic production.
  b. Experimental values are then normalized to the mean value of the control group (relative units *vs.* control).

## Expected outcomes

H_2_S is traditionally recognized as a toxic gas; however, it is now well established that H_2_S also acts as an important biological signalling molecule with beneficial physiological effects. Despite its relevance, the lack of optimal and standardized methods for reliable H_2_S detection has limited its study. To address this, we describe two complementary approaches to investigate H_2_S content: Workflow 1, which detects H_2_S levels in solution, and Workflow 2, which measures cumulative H_2_S production.

Using Workflow 1, we evaluate the non-enzymatic production of H_2_S by DAD and DAT using an ion-selective microelectrode (ArrowH_2_S™). DAT alone produces significant H_2_S levels in solution, whereas DAD alone does not. The addition of GSH enhances H_2_S production by both compounds. GSH is essential for DAD-mediated H_2_S generation, whereas DAT shows only a modest increase. Liver lysates do not alter H_2_S levels (Figure 4).

Using Workflow 2, we evaluated the enzymatic production of liver of mice fed with a standard healthy diet supplemented with DAD or DAT using lead acetate/lead sulfide paper method (Figure 6A). The final concentrations were PLP 1 mM and L-cysteine 10 mM. Measurements were obtained from protein extracts (35 μg protein/well) prepared from livers of mice fed with either DAD or DAT. Liver extracts isolated from mice fed with DAD and DAT exhibited reduced enzymatic H_2_S production. Furthermore, we investigated whether naturally occurring sulfur-containing compounds enhance non-enzymatic H_2_S production over time using the lead acetate/lead sulfide method (Figures 6B and 6C). For this purpose, in Figure 6B, we tested three garlic-derived compounds, DAM (0–1 mM), DAD (0–1 mM) and DAT (0–1 mM). The final GSH concentration in the assay was 50 mM. Our results showed that DAM did not produce H_2_S, while DAD and DAT promoted H_2_S production, being DAT the higher H_2_S producer. Liver lysates enhanced the cumulative production of H_2_S from DAD and DAT (Figure 6B). We also performed assays in the presence of AOAA which inhibits PLP-dependent catalysis (Figure 6C). The final concentration GSH concentration was 50 mM and AOAA 2 mM, respectively. H_2_S generation from DAD and DAT was unaffected by AOAA, indicating that DAD and DAT-derived H_2_S production is not PLP dependent (Figure 6C). In conclusion, the combined use of these two complementary workflows provides a reliable approach to assess both non-enzymatic and enzymatic H_2_S production, enabling a more accurate assessment of H_2_S generation by garlic-derived compounds in biological systems.

## Limitations

Workflow 1: In our experience, H_2_S levels are undetectable in cell supernatants, likely because levels are under the threshold of detection.

Workflow 2: The lead acetate method is a semi-quantitative assay that reflects H_2_S production capacity relative to controls, rather than absolute H_2_S levels. Optimization of lead acetate paper exposure timing is challenging. Short exposure timing may lead to undetectable H_2_S generation. Long exposure timing may lead to saturation of the lead acetate paper, impeding comparative evaluations among samples. Likewise, due to the different rates of sulfide off gassing between simple sulfide salt standards and those of the enzymatic/chemically catalyzed reactions that release sulfide, accurate determination of absolute sulfide values is difficult.

## Troubleshooting

### Problem 1

The ArrowH_2_S™ micro-sensing electrode becomes highly unstable unless it is completely filled with KCl (4 M) saturated with AgCl solution. Related to “before you begin” section.

### Potential solution

Refill the electrode daily, carefully removing any bubbles, and ensuring it is properly sealed with a sticker before performing measurements.

### Problem 2

The ArrowH_2_S™ micro-sensing electrode is prone to protein contamination after measuring biological samples, affecting accuracy and stability of determinations. Related to step 4.

### Potential solution

Clean the ArrowH_2_S™ micro-sensing electrode with a protein removal solution before measurements, or at least once per week, to eliminate any proteins adhered to the H_2_S sensing electrode. First, rinse the electrode with distilled water. Then immerse the electrode for 5 min in protein cleaning solution in a 15-mL Falcon tube. After this, rinse the electrode with distilled water to ensure that all traces of the cleaning solution are removed.

### Problem 3

Disrupted formation of the H_2_S production circles. This is caused because the paper is not perfectly attached to the plate and/or shifts during the procedure. Related to step 8.

### Potential solution

Place the lead acetate paper on the plate, cover it with the lid of another plate upside down, and tape to secure the paper completely to the plate or place a heavy weight on top of the upside down plate cover.

### Problem 4

Lead sulfide circles go beyond the edge of the well or artefacts are found in the lead acetate paper after the assay. Related to step 12.

### Potential solution

Samples with high H_2_S production that could affect adjacent wells, leave empty wells between samples fill these wells with PBS. Before placing the blotting paper, make sure the plate rims of the wells are thoroughly dried to prevent interference.

### Problem 5

A key challenge is that if the blotting paper is incubated at 37 °C for too short or too long, leading to no circles or saturated circles, impeding proper image analysis. Related to step 12.

### Potential solution

Prepare several identical plates and remove the paper at different time points. This approach allows determination of the optimal time for image acquisition. Three technical replicates are also recommended to ensure reliability.

## Resource availability

### Lead contact

Further information and requests for resources and reagents should be directed to and will be fulfilled by the lead contact, Alejandro Martín-Montalvo (alejandro.martinmontalvo@cabimer.es).

### Technical contact

For technical specifics on executing the protocol, María Ángeles Cáliz-Molina (maria.caliz@cabimer.es) and Inmaculada Pino-Pérez (inmaculada.pino@cabimer.es) will provide support to ensure its correct implementation.

### Materials availability

This study did not generate new unique reagents or materials.

### Data and code availability

This protocol does not report any original code. Part of the data presented is derived from the same experiments as reported in Caliz-Molina, MA et al.^1^

## Acknowledgments

This work was funded by the 10.13039/501100004837Ministerio de Ciencia e Innovación/AEI/10.13039/501100011033 and FEDER, a way of making Europe; 10.13039/100011997FSE invest in your future (PID2021-123965OB-100 and PID2024-156080OB-I00 to A.M.-M., FPU19/05468 to M.A.C.-M., and FPU21/01338 to I.P.-P.); and 10.13039/100020230Junta de Andalucía of public funding, in the 2024 call of the 10.13039/501100023731Consejería de Salud y Consumo for the financing of research, development, and innovation (R&D&I) in Biomedicine and Health Sciences in Andalusia (PI-003502024 to A.M.-M). We acknowledge the support of the Basic Research Group of the 10.13039/100022835Spanish Society of Diabetes, 10.13039/501100013941CIBERDEM and the Conexión COMETA of 10.13039/501100003339CSIC.

## Author contributions

M.A.C.-M. and I.P.-P. performed the experiments. All authors developed the conceptual framework, wrote the manuscript, discussed the results, and commented on the manuscript.

## Declaration of interests

The authors declare no competing interests.
